# Score prediction of anastomotic leak in colorectal surgery: a systematic review

**DOI:** 10.1007/s00464-024-10705-1

**Published:** 2024-02-28

**Authors:** Alexis Litchinko, Nicolas Buchs, Alexandre Balaphas, Christian Toso, Emilie Liot, Guillaume Meurette, Frédéric Ris, Jeremy Meyer

**Affiliations:** 1 Division of Digestive Surgery, University Hospitals of Geneva, Rue Gabrielle-Perret-Gentil 4, 1211 Geneva 14, Switzerland; 2 Medical School, University of Geneva, Rue Michel-Servet 1, 1205 Geneva, Switzerland

**Keywords:** Anastomotic leak, Colorectal surgery, Score, Risk assessment, Postoperative complications

## Abstract

**Objective:**

Predicting the risk of anastomotic leak (AL) is of importance when defining the optimal surgical strategy in colorectal surgery. Our objective was to perform a systematic review of existing scores in the field.

**Methods:**

We followed the PRISMA checklist (S1 Checklist). Medline, Cochrane Central and Embase were searched for observational studies reporting on scores predicting AL after the creation of a colorectal anastomosis. Studies reporting only validation of existing scores and/or scores based on post-operative variables were excluded. PRISMA 2020 recommendations were followed. Qualitative analysis was performed.

**Results:**

Eight hundred articles were identified. Seven hundred and ninety-one articles were excluded after title/abstract and full-text screening, leaving nine studies for analysis. Scores notably included the Colon Leakage Score, the modified Colon Leakage Score, the REAL score, www.anastomoticleak.com and the PROCOLE score. Four studies (44.4%) included more than 1.000 patients and one extracted data from existing studies (meta-analysis of risk factors). Scores included the following pre-operative variables: age (44.4%), sex (77.8%), ASA score (66.6%), BMI (33.3%), diabetes (22.2%), respiratory comorbidity (22.2%), cardiovascular comorbidity (11.1%), liver comorbidity (11.1%), weight loss (11.1%), smoking (33.3%), alcohol consumption (33.3%), steroid consumption (33.3%), neo-adjuvant treatment (44.9%), anticoagulation (11.1%), hematocrit concentration (22.2%), total proteins concentration (11.1%), white blood cell count (11.1%), albumin concentration (11.1%), distance from the anal verge (77.8%), number of hospital beds (11.1%), pre-operative bowel preparation (11.1%) and indication for surgery (11.1%). Scores included the following peri-operative variables: emergency surgery (22.2%), surgical approach (22.2%), duration of surgery (66.6%), blood loss/transfusion (55.6%), additional procedure (33.3%), operative complication (22.2%), wound contamination class (1.11%), mechanical anastomosis (1.11%) and experience of the surgeon (11.1%). Five studies (55.6%) reported the area under the curve (AUC) of the scores, and four (44.4%) included a validation set.

**Conclusion:**

Existing scores are heterogeneous in the identification of pre-operative variables allowing predicting AL. A majority of scores was established from small cohorts of patients which, considering the low incidence of AL, might lead to miss potential predictors of AL. AUC is seldom reported. We recommend that new scores to predict the risk of AL in colorectal surgery to be based on large cohorts of patients, to include a validation set and to report the AUC.

**Supplementary Information:**

The online version contains supplementary material available at 10.1007/s00464-024-10705-1.

Anastomotic leak (AL) is a dreaded complication in colorectal surgery. In the 2018 European Society of Coloproctology (ESCP) audit, AL was clinically or radiologically proven in 7.3% of patients who had an anastomosis in the left colon, sigmoid or rectum [1]. In another recent retrospective study, Rencuzogullari et al. [2] reported an incidence of AL of 3.2% in 10.392 patients who underwent elective colectomy and/or anterior resection. More specifically, in rectal surgery, the incidence of AL reached 24.7% in the randomized controlled GRECCAR 5 trial [3].

AL has serious consequences on perioperative morbidity and mortality, and on long-term survival after oncologic surgery. For instance, AL is associated with increased incidence of local recurrence and reduced long-term and overall survival in patients with colorectal cancer [4]. Moreover, the economic burden caused by AL significantly impairs healthcare systems budgets. In a retrospective study including 600 hospitals in the USA, Hammond et al. reported that AL led to an increase of the economic burden by a factor ranging between 0.6 and 1.9 for 30-day re-admission, postoperative infection, length of stay and hospital costs [5]. Moreover, several prophylactic measures are routinely taken to reduce the clinical impact of potential AL in patients with anastomoses at higher risk of AL. These measures may increase the morbidity of colorectal surgery as well as impairing patient’s quality of life. For instance, subperitoneal colorectal anastomoses after low anterior resection are routinely diverted with a temporary defunctioning loop ileostomy, which leads to an increased risk of rehospitalization for dehydration due to acute renal failure caused by high ileostomy output, alteration of quality of life, troubles with stoma management and costs associated with a second surgery to close the ileostomy [6, 7].

To decrease the incidence of AL, attempts have been made to identify pre-operative risk factors for AL [8–11]. Among measures targeting controllable risk factors, we can mention the use of fluorescence angiography [12–14], mechanical bowel preparation, preoperative antibiotics [15], preoperative iron [16] and specific perioperative program like the Enhanced Recovery After Surgery (ERAS) [17].

In order to reduce the risk of AL, identify patients at risk of AL and help in the surgical decision-making, several scores allowing to predict the risk of AL have been developed over the years. As their ability to predict AL has not been compared in the recent literature, our objective was to perform a systematic review of existing scores and to identify important factors that could predict AL in colorectal surgery.

## Materials and methods

**Fig. 1:**
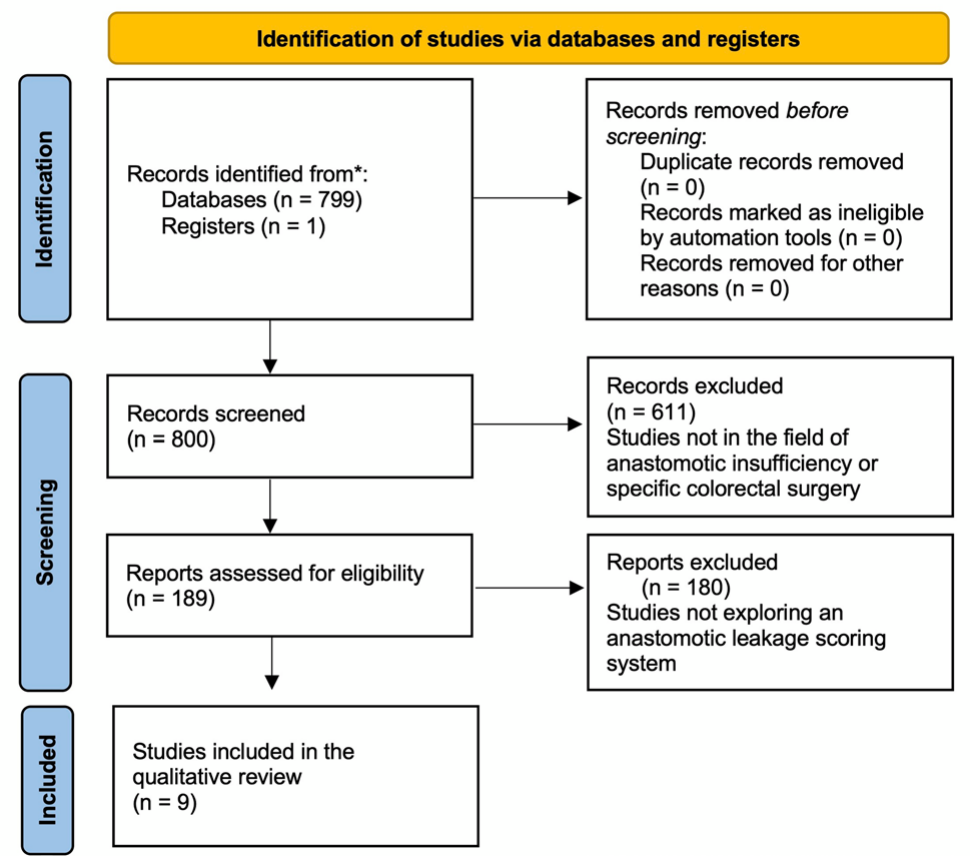
Preferred Reporting Items for Systematic Review and Meta-analyses (PRISMA) flowchart.

The systematic review was performed according to the Preferred Reporting Items for Systematic Reviews and Meta-Analyses (PRISMA) 2020 guidelines [18] (Fig. 1). An ethics statement is not applicable because this study is based exclusively on published literature.

### Literature search strategy

MEDLINE, Embase and the Cochrane Central Register of Controlled Trials were searched on the 01.04.2021 without time limit for original studies reporting on scores allowing to predict the occurrence of AL in colorectal surgery. Databases like ClinicalTrials.gov and the WHO International Clinical Trials Registry Platform were explored, aiming to discover pertinent studies that may not have been cataloged in the main databases. Only studies in English were included. The search strategy is reported in Table 1. Additional studies were also identified through Google search and manual search of the reference list of included studies.

**Table 1:** Search strategy

Database	Search build	Occurrences
MEDLINE	“(Anastomotic leakage[Title/Abstract] OR anastomotic leak[Title/Abstract]) AND (score[Title/Abstract])”	583 (14)
EMBASE	‘Anastomosis leakage’:ti,ab,kw AND score:ti,ab,kw	34 (0)
COCHRANE CENTRAL	“Score” in Title Abstract Keyword AND “anastomosis” in Title Abstract Keyword AND “leakage” in Title Abstract Keyword -	182 (0)
Other sources		1

### Inclusion and exclusion criteria

Original studies reporting on scores allowing predicting the occurrence of AL in colorectal surgery were considered for inclusion. Conference abstracts, letters to the editor and systematic reviews were excluded after title and abstract screening. Articles with multiple anastomoses performed during the same procedure were excluded. Studies not reporting on the variables included in the score were also excluded. Two independent reviewers (AL, JM) carried out the systematic review. Discrepancies were solved by a third author (FR) (Table 2).

**Table 2:** Included studies

Study	Year	Country	Score name	Patients included, *n*	Type of surgery	AL, %
McKenna et al. [24]	2019	USA	–	38,475	Left-sided colorectal surgery^a^	3.3%
Yang et al. [25]	2019	South Korea	Modified Colon Leakage Score	566	Left-sided colorectal surgery^a^	4.1%
Rencyzogullari et al. [2]	2017	USA	–	10,392	All colorectal surgery^b^	3.2%
Kim et al. [26]	2017	Korea	–	736	Low anterior resection	8.8%
Rojas-Machado et al. [19]	2016	Spain	PROCOLE	N/A	All colorectal surgery^b^	N/A
Frasson et al. [20]	2015	Spain	–	3193	All colorectal surgery^b^	8.7%
Pasic et al. [21]	2013	Bosnia & Herzegovina	–	159	All colorectal surgery^b^	14.5%
Dekker et al. [22]	2011	Netherlands	Colon Leakage Score	139	Left-sided colorectal surgery^a^	8.7%
Arezzo et al. [23]	2011	Italy	REAL (Rectal Anastomotic Leak)	9735	Low anterior resection	9.7%

^a^Left hemicolectomy, sigmoid colectomy, high anterior resection and low anterior resection

^b^Ileocecal resection, right hemicolectomy, left hemicolectomy, sigmoid colectomy, high anterior resection and low anterior resection

### Data extraction

Two authors (AL, JM) performed data extraction from included publications. The following variables were extracted: first author, year of publication, country of the study, period of inclusion, methodological information, demographic characteristics of selected patients, type of surgery performed, type of anastomosis, definition of AL, presence of a score, variables assessed by the score and scientific validation of the scores.

## Results

### Inclusion process

The literature search strategy identified 800 eligible studies. Five-hundred and eighty-three studies were identified in Medline, 34 in Embase and 182 in the Cochrane Central Register of Controlled Trials. Six-hundred and eleven articles were excluded after title and abstract screening. From the 188 full text articles assessed for eligibility, 179 studies were removed for not fulfilling inclusion criteria and/or meeting at least one of the exclusion criteria. From the nine studies left, all fulfilled the inclusion criteria, respected the exclusion criteria and were eligible for the qualitative analysis (Fig. 1).

### Characteristics of included studies

Five studies were performed in Europe [19–23], two in the United States [2, 24] and two in Korea [25, 26]. First patient recruitment began in 2000. Four studies were monocentric [21, 22, 25, 26], four were multicentric [2, 20, 23, 24] and one used original data from other publications [19]. Scores included the Colon Leakage Score [22], the modified Colon Leakage Score [25], the REAL score [23], www.anastomoticleak.com [27] and the PROCOLE score [19]. Four studies (44.4%) included more than 1000 patients [2, 20, 23, 24]. Four studies included all types of colorectal surgery procedures [2, 19–21] and three only included patients with left-colorectal surgery with left hemicolectomy, sigmoid colectomy, high anterior resection and low anterior resection. [22, 24, 25]. Two studies only included colorectal anastomosis with low anterior resection [23, 26]. A clear and specific definition of AL was detailed in six studies (66.6%) [2, 20–22, 25, 26]. The incidence of AL ranged from 3.2 to 14.5% among included studies [2, 21].

### Variables included in the scores

Studies included the following pre-operative variables in their scores for prediction of AL: distance from the anal verge (eight studies, 88.8%) (2, 20, 22–27), sex (seven studies, 77.8%) (2, 20–21, 23–25, 27), ASA score (six studies, 66.6%) (2, 20, 22–23, 25–26), age (four studies, 44.4%) (20, 23, 25, 27), neo-adjuvant treatment (four studies, 44.9%) (20, 23–25), BMI (three studies, 33.3%) (20, 21, 23), smoking (three studies, 33.3%) (20, 23, 25), alcohol consumption (three studies, 33.3%) (20, 23, 26), steroid consumption (three studies, 33.3%) (2, 20, 26), diabetes (two studies, 22.2%) (2, 20), respiratory comorbidity (two studies, 22.2%) (2, 20), hematocrit (two studies, 22.2%) (20, 25), anticoagulant medication (one study, 11.1%) (21), cardiovascular comorbidity (one study, 11.1%) (20), liver comorbidity (one study, 11.1%) (20), weight loss (one study, 11.1%) (2), total proteins (one study, 11.1%) (20), WBC (one study, 11.1%) (20), albumin (one study, 11.1%) (20), hospital size (one study, 11.1%) (21), bowel preparation (one study, 11.1%) (25) and indication for surgery (one study, 11.1%) (20) (Table 3).

**Table 3:**
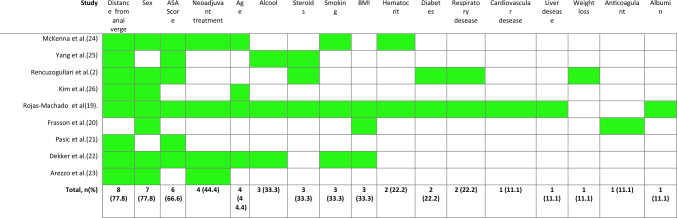
Preoperative variables selected in scores

The green color means that the value is included in the score. Absence of color (white) means that it is not included in the score

Studies included the following peri-operative variables in their scores for prediction of AL: duration of surgery (six studies, 66.6%) (2, 20, 22–23, 25, 27), blood loss/transfusion (five studies, 55.6%) (20, 22–24, 27), additional procedure (three studies, 33.3%) (20, 23, 26), emergency surgery (two studies, 22.2%) (20, 23), operative complication (two studies, 22.2%) (20–21), surgical approach (two studies, 22.2%) (2, 25), wound contamination class (one study, 1.11%) (21), mechanical anastomosis (one study, 1.11%) (20) and experience of the surgeon (one study, 11.1%) (20) were specifically used (Table 4).

**Table 4:**
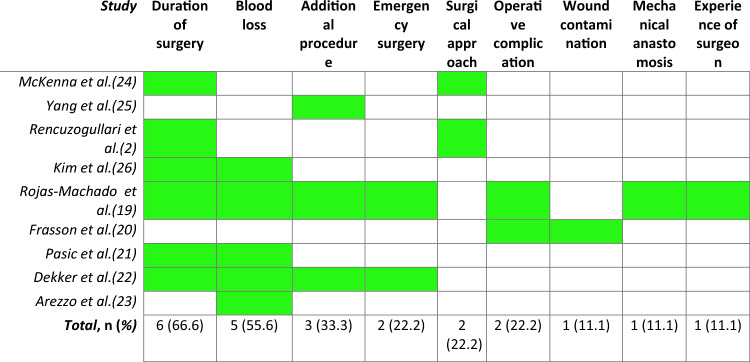
Intraoperative variables selected in scores

The green color means that the value is included in the score. Absence of color (white) means that it is not included in the score

### Discriminating ability of scores

Six studies reported the area under the ROC curve (AUC) [19, 21–23, 25, 26]. Arezzo et al. [23] with the REAL score (Rectal Anastomotic Leak) reported an AUC of 0.597 (95% CI 0.585–0.608). Pasic et al. [21] announced an AUC of 0.973 (95% CI 0.934–0.992). Yang et al. [25] with the mCLS (modified Colon Leakage Score) outlined an AUC of 0.831 (95% CI 0.767–0.896). Rojas-Machado et al. [19] with the PROCOLE (Prognostic Colorectal Leakage) score reported an AUC of 0.82 (95% CI 0.75, 0.89). Kim et al. [26] documented an AUC of 0.753 (95% CI 0.690–0.816). Finally, Dekker et al. [22] with the CLS (Colon Leakage Score) published an AUC of 0.95 (95% CI 0.89–1.00) (Table 5).

**Table 5:** Selected studies with AUC, CI, *p*-value and validation cohort with size of validation population if present

Study	Year	Score name	AUC (95% CI)	p-value	Validation cohort	Size of validation population
McKenna et al. [24]	2019	–	–	N/A	No	–
Yang et al. [25]	2019	Modified Colon Leakage Score	0.831 (0.767–0.896)	*p* = 0.008	Yes	170
Rencyzogullari et al. [2]	2017	–	–	N/A	No	–
Kim et al. [26]	2017	–	0.753 (0.690–0.816)	N/A	No	–
Rojas-Machado et al. [19]	2016	PROCOLE (Prognostic Colorectal Leakage)	0.82 (0.75–0.89)	SE = 0.04	No	–
Frasson et al. [20]	2015	www.anastomoticleak.com	–	N/A	No	–
Pasic et al. [21]	2013	–	0.973 (0.934–0.992)	*p* < 0.001	Yes	40
Dekker et al. [22]	2011	Colon Leakage Score	0.95 (0.89–1.00)	*p* < 0.01	Yes	121 (304)
Arezzo et al. [23]	2011	REAL (Rectal Anastomotic Leak)	0.597 (0.585–0.608)	*p* < 0.0001	Yes	2921

*SE* standard error

### Validation of scores

Four scores (44.4%) (22–24, 26) included a validation set. For instance, Yang et al. [25] used a 170 patients validation cohort and the predictive performance of the modified Colon Leakage Score was similar in the training and in the validation cohorts (AUC: 0.838 vs 0.803, *p* = 0.724). Pasic et al. [21] had a small size validation cohort with only 40 patients, with an AUC for the score of 1.0. Dekker et al. [22] reported a 121 patients validation cohort and later performed a validation study to evaluate the effectiveness of the AL prediction [28]; Yu et al. evaluate the clinical utility of the colon leakage score (CLS) in predicting the risk of AL and reported an AUC of 0.965 (IC: 0.913–1.00). Arezzo et al. [23] proposed a large validation cohort with nearly 3000 patients with 77.8% of sensitivity and 35.2% specificity for determining the risk of AL.

## Discussion

In this systematic review, we identified nine scoring systems that predict AL in colorectal surgery. These scores were derived from a comprehensive analysis of 63,395 pooled patients, including a validation subset of 3252 patients.

The currently available scoring systems for evaluating AL exhibit significant limitations in terms of their comprehensiveness and reliability. These scoring systems, designed to assess the occurrence of AL are characterized by incomplete representation of relevant factors and an overall lack of consistent predictive accuracy. The presence of heterogeneity among these scoring systems underscores the absence of a clear and standardized approach to accurately predict AL. Variations in criteria, variables, and thresholds employed by different scoring systems contribute to the heterogeneous nature of the available scoring methodologies.

Among the various scores, the distance from the anal verge emerged as the most consistent predictor of AL, being supported by eight studies (88.8%) (2, 20, 22–27). For instance, inadequate vascularization of the colonic conduit following division of the inferior mesenteric artery and tension on the anastomosis due to insufficient mobilization of the splenic flexure can explain AL and may be more prevalent in lower tumors. Additionally, some studies highlighted the potential of arterial calcification as being a predictor of AL [29]. Therefore, this variable should potentially be evaluated in future scoring systems through pre-operative imaging.

Moreover, we note that several predictors identified in the scores were associated with factors influencing wound healing, such as age, albumin concentration, hemoglobin concentration, smoking, steroid use, diabetes, anticoagulation, immunosuppressant use, active neoplasia, and chronic conditions. Therefore, we recommend that future scoring systems consider incorporating additional factors related to wound healing, such as pre-albumin concentration, underlying vascular disease, tumor size, and the use of intraoperative angiography. Rectal tumors, particularly those in the lower third of the rectum, are associated with higher rates of leakage compared to colonic tumors. This increased risk can be attributed to the technical challenges and reduced blood supply in this region. Additionally, the necessity for neoadjuvant therapy in rectal cancer, unlike in many colon cancers, further exacerbates this risk. Furthermore, the type of anastomosis performed, whether it is a coloanal, colorectal, or colocolic anastomosis, is directly determined by the tumor location and consequently influences leakage rates. Coloanal anastomoses, often required for lower rectal tumors, have a higher leakage risk due to the complexity of the procedure and the lower blood supply in the anorectal region. Understanding these correlations is crucial for surgical planning and patient counseling, emphasizing the need for tailored surgical approaches based on tumor location to minimize the risk of anastomotic leakage.

Sex was also identified as a predictor of AL in seven studies (77.8%) (2, 20–21, 23–25, 27). However, this finding may be confounded by factors like dietary habits, smoking history, and alcohol consumption, all of which can impair anastomotic healing [30]. Consequently, it is crucial to conduct multivariate analysis to account for confounding variables.

However, among the studies included in our review, only five out of nine performed multivariate analysis [2, 20, 21, 23, 26]. Developing a multivariable statistical model to estimate the risk of AL is a complex task due to the involvement of numerous variables. Optimal identification of predictors primarily relies on sample size and the incidence of AL as the outcome. The incidence of AL in colorectal surgery ranged from 3.2 [2] to 14.5% [21] in the studies included in our review. Given the potentially low incidence of AL, the identification of predictors, especially when multiple factors are involved, would require a substantial number of patients. Surprisingly, some scoring systems were based on cohorts with a small number of patients, potentially leading to the omission of important predictors, like type II statistical error. For example, the Colon Leakage Score, developed based on a cohort of only 139 patients with 12 AL cases, may introduce selection bias and inadequately capture evidence of AL.

Furthermore, and probably the most importance, during the creation of scoring systems, selection bias inevitably occurs since the patients at higher risk of AL are likely excluded from receiving an anastomosis. For instance, most of the scores evaluated were based on cohorts of elective surgery patients and did not consider variables such as vasoactive drugs, hemodynamic status, or abdominal cavity contamination, which are relevant in emergency surgery scenarios where the decision to perform an anastomosis is critical [31, 32]. Applying these scores routinely in emergency situations may not be harmless, and further studies are needed to evaluate and develop scores specifically for emergency settings. Finally, only six studies provided detailed statistical validity results [19, 21–23, 25, 26], including evidence of the area under the curve (AUC), which is essential for validating the strength of a score. Only four scores (44.4%) (22–24, 26) included a validation set to demonstrate the reliability of the score. External validation is crucial in score building studies, and it is important to plan for a validation cohort from the outset.

Lastly, the presence of a diverting stoma may influence the incidence of AL, as some cases may remain subclinical and undetected.

To conclude, the development of scoring systems for predicting AL in colorectal surgery requires careful consideration of multiple factors and adequate sample sizes. Future studies should address the limitations identified in this review and focus on emergency surgery scenarios, provide detailed information on the anastomosis technique, incorporate nutritional and anemia factors, and evaluate liver function as a predictor. External validation and statistical reliability assessments are essential in the validation process of scoring systems.

## Conclusion

Our findings reveal significant limitations in the existing scoring systems for anastomotic leakage (AL), primarily concerning the variables considered, as well as the definition and diagnostic methods used for AL. Considering these limitations, we strongly advocate for the development of a new scoring system that addresses these issues.

To overcome the limitations, we propose that a prospective cohort study be conducted, employing more accurate methods for determining AL, such as contrast enema or endoscopy, to actively detect cases. Additionally, it is crucial to systematically collect all variables that influence AL to ensure comprehensive data collection.

Furthermore, in order to enhance the accuracy and applicability of the new scoring system, a homogeneous cohort of patients should be utilized, avoiding subgroup analyses based solely on anastomosis location (right, left, or precise rectal localization) or whether the resection is oncological or not. This approach will enable a more comprehensive understanding of the predictors of AL across the spectrum of colorectal surgery.

### Supplementary Information

Below is the link to the electronic supplementary material.

Supplementary file1 (DOCX 31 KB)

Supplementary file2 (DOCX 25 KB)
